# Could dietary restrictions affect periodontal disease? A systematic review

**DOI:** 10.1007/s00784-023-05052-9

**Published:** 2023-05-18

**Authors:** Giuseppe Mainas, Pasquale Santamaria, Mark Ide, Valter Longo, Manlio Vinciguerra, Jose Nart, Luigi Nibali

**Affiliations:** 1 Periodontology Unit, Centre for Host Microbiome Interactions, Faculty of Dentistry, Oral & Craniofacial Sciences, King’s College London, London, UK; 2 Longevity Institute, Leonard Davis School of Gerontology and Department of Biological Sciences, University of Southern California, Los Angeles, CA USA; 3 Liverpool Center for Cardiovascular Science, Liverpool John Moores University, Liverpool, UK; 4 Department of Periodontology, Universitat Internacional de Catalunya, Barcelona, Spain

**Keywords:** Periodontitis, Diet, Caloric restriction, Fasting, Inflammation, Systematic review

## Abstract

**Objective:**

This review aimed at evaluating the possible benefits that caloric restriction (CR) may provide to periodontal disease progression and response to treatment.

**Material and methods:**

Electronic search on Medline, Embase and Cochrane, and manual search were performed to identify pre-clinical and on human studies reporting the consequences of CR on clinical and inflammatory parameters related to periodontitis. Newcastle Ottawa System and SYRCLE scale were used to assess the risk of bias.

**Results:**

Four thousand nine hundred eighty articles were initially screened, and a total of 6 articles were finally included, consisting of 4 animal studies and 2 studies in humans. Due to the limited number of studies and heterogeneity of the data, results were presented in descriptive analyses. All studies showed that, compared to the normal (ad libitum) diet, CR might have the potential to reduce the local and systemic hyper-inflammatory state as well as disease progression in periodontal patients.

**Conclusions:**

Within the existing limitations, this review highlights that CR showed some improvements in the periodontal condition by reducing the local and systemic inflammation related to the periodontitis and by improving clinical parameters. However, the results should be interpreted with caution since robust research such as randomized clinical trials is still missing.

**Clinical relevance:**

This review shows that some dietary/caloric restrictions approaches may have the potential to improve periodontal conditions and, in addition, highlights a need for human studies with a robust methodology in order to draw stronger evidence-based conclusions.

**Supplementary Information:**

The online version contains supplementary material available at 10.1007/s00784-023-05052-9.

## Introduction

Periodontitis is a microbially driven host-mediated disease that leads to loss of periodontal attachment and bone [1]. At first stage, gingival inflammation (or gingivitis) is caused by bacterial biofilm formation. If left untreated, it may progress to destructive periodontitis. This step depends on microbial dysbiosis in response to nutrients from gingival inflammatory and tissue breakdown products that favour some microbial species, and on anti-bacterial mechanisms that try to contain the microbial challenge within the gingival sulcus [1].

Nutrition is intended as the process of taking in food and its use for growth, metabolism and repair [2]. While nutrition is a fundamental process for all physiological functions, pre-clinical studies reported that caloric restriction, generally intended as a lowering of calorie intake by 20% or more below the level needed to maintain a normal body weight without causing malnourishment, improved the overall health, function and longevity of rhesus monkeys [3, 4].

The effects of nutrition in maintaining health and in the predisposition to periodontal disease has been gradually emerging in several studies that reported effects such as gingival bleeding reduction [5], reduced gingival inflammation [6] and reduced tooth loss [7].

Several human and animal investigations have also been published concerning the possible effects of nutrition on management of periodontal disease.

A recent systematic review of animal models concluded that macronutrients that have any effect on oxidative stress or immune system (i.e., n-3 fatty acid) seem to be important for the prevention or treatment of periodontal disease [8]. A German team reported that a diet consisting of a low carbohydrates intake, rich in omega 3-fatty acids and fibre, higher vitamins C and D values, low animal proteins and rich in nitrate-containing plants might lead to a significant reduction in periodontal and gingival inflammation [9, 10], even though serological inflammatory parameters and the subgingival microbiome seemed to be unaffected [10]. More recently, a study found that there was no overall association between either Western dietary patterns (characterized by a higher intake of processed meat, red meat, butter, high-fat dairy products, eggs and refined grains) or prudent dietary patterns (characterized by a higher intake of vegetables, fruit, legumes, whole grains, fish and poultry) and periodontitis, although if only obese individuals were studied, a Western diet was related to a higher risk of periodontitis [11]. Nevertheless, a systematic review and meta-analysis of observational studies reported that Western diet, high sugar intake and dairy products did not significantly affected the prevalence of periodontitis, whereas, very interestingly, vitamin C low intake was positively associated with a higher risk of periodontal disease [12]. Another very recent investigation, using a dietary inflammatory index, showed that consuming a pro-inflammatory diet was associated with periodontal disease in the US general adult population. It was observed that an anti-inflammatory diet would be more beneficial in adults > 60 years old males [13].

Furthermore, different approaches including fasting diets have been used over the years with promising results in terms of longevity and health span [14]. Two types of regimens have been reported in literature: intermittent fasting (IF) and periodic fasting (PF). In brief, IF might be either a complete fasting every day or an alternation of hours (in the same day)/days of fasting with hours (in the same day)/days of ad libitum eating period. In contrast, PF might consist of a period of water-only fasting followed an ad libitum eating period of at least 7 days or a caloric intake reduction for 4–7 consecutive days followed by a normal refeeding period once a month [14].

Up to date, only few studies investigated the possible effect of a caloric restriction regimen on periodontal disease, and the results were controversial in terms of systemic outcomes (i.e. inflammation) and clinical parameters [15, 16].

However, to our knowledge, no systematic reviews have been published to date which summarize the evidence about the potential effect of caloric restriction on periodontal disease. Therefore, the aims of the present systematic review were to (i) assess if restriction of caloric intake may have an effect on periodontal disease and response to treatment and (ii) summarize the quality of the existing evidence of caloric restriction and periodontitis.

## Material and methods

### Protocol registration

The present review was registered in PROSPERO (International Prospective Register of Systematic Reviews hosted by the National Institute for Health Research, University of York, Centre for Reviews and Dissemination—#CRD42021268785).

### Reporting format

This systematic analysis was performed according to the Preferred Reporting Items for Systematic Review and Meta‐Analyses (PRISMA) statement [17, 18].

### Focused question

Does a restriction of caloric intake have an effect on periodontal disease and treatment response (in terms of clinical measurements and measures of inflammation)?

### Eligibility: inclusion and exclusion criteria

#### P(E)ICOS

- Population: patients with measures of periodontal disease
- Exposure/intervention: diets with caloric restriction
- Control: patients continuing their regular diet or a diet with no caloric restriction
- Outcomes: inflammatory markers, clinical variables (periodontal parameters) and patient-reported outcome measurements (PROMs)
- Study design: randomized controlled trials, cross-sectional studies, case-control studies, longitudinal cohort studies (retrospective or prospective), and animal studies reporting data on (i) periodontal disease diagnosis (at baseline and if applicable post-intervention) and comparison between two groups, including one with caloric restriction and one with normal diet (or no caloric restriction), were included.
  Reviews, case reports and studies which included less than 10 participants were excluded.

### Identifying research evidence

Electronic literature searches were conducted independently by two authors (GM and PS) through Medline, Embase and Cochrane Library Database, including studies published up to November 15, 2022.

The following research strategy was used: (((hypocaloric diet (keywords) OR caloric restriction (MeSH terms) OR nutrition (keywords) OR diet (MeSH terms) OR fasting (MeSH terms)) OR dietary intervention (keywords) AND ((periodontitis (MeSH terms) OR periodontal diseases (MeSH terms) OR gingivitis (MeSH terms) OR periodontal patients (keywords) OR periodontal inflammation (keywords) OR gingival (keywords)).

The search was complemented by an Open Grey search and by conducting a manual search (including *Journal of Dental Research, Journal of Clinical Periodontology, Journal of Periodontology* and *Journal of Periodontal Research* (from 2000).

No language restriction was included in the search. Among published literature, peer-reviewed studies, reports and book chapters were screened. Narrative or systematic reviews on the topic were searched in order to identify suitable papers.

### Data collection process

Study selection was conducted by two independent reviewers (GM and PS) in the following stages:

1. Initial screening of potentially-suitable titles and abstracts against the inclusion criteria to identify potentially relevant papers.
2. Screening of the full papers identified as possibly relevant in the initial screening.

Any papers that did not meet the inclusion criteria were excluded. After the full text screening (steps 1 and 2), the two reviewers compared the studies included by at least one of them and joined them all into a final database.

### Data extraction

Data were independently extracted by two independent reviewers (GM and PS). A data extraction form was used for each study in order to extract relevant data (mentioned below) to be included in this review:

- Study design
- Number of patients included
- Population demographic (e.g., age, gender, ethnicity)
- Setting (country, hospital, university, community, others)
- Definition and diagnosis of periodontal disease/health and of periodontal progression
- Definition of dietary intervention
- Control group
- Periodontal intervention (if any)
- Which variables were measured before and after intervention/control
- Outcomes of the intervention
- Funding
- Conflict of interest

### Definition of periodontal disease/periodontal health

In studies published after 2017, classification of periodontitis according to the “2017 World Workshop” including Staging and Grading [1] was considered ideal, whereas for studies prior to 2017, the 1999 Classification of Periodontal Disease (AAP) [19] or previous classifications (“Proceeding of the World Workshop in Clinical Periodontics (AAP) 1989) [20] should have been used. During data extraction, it was indicated if a clear definition of periodontal disease (according classifications above) or not was provided.

### Definition of caloric restriction

Caloric restriction is ideally defined as a reduction of average daily caloric intake, between 10 and 40%, without malnutrition and without affecting the intake of essential nutrients like vitamins and minerals [21]. However, other definitions provided by the authors were considered and reported.

### Methodological quality assessment

The Newcastle Ottawa System (NOS) protocol (Newcastle Ottawa Scale http://www.ohri.ca/programs/clinical_epidemiology/oxford.htm) was followed for assessing the risk of bias in human studies, whereas, for animal studies, the Systematic Review Center for Laboratory Animal Experimentation (SYRCLE) tool24 [22] was used. The assessment was independently performed by two reviewers (G.M. and P.S.).

### Data analysis and synthesis of results

Data were presented descriptively. Two separate data syntheses were provided for animal and human studies, respectively. Each outcome of the included studies was showed in summary tables.

## Results

### Study selection

**Fig. 1:**
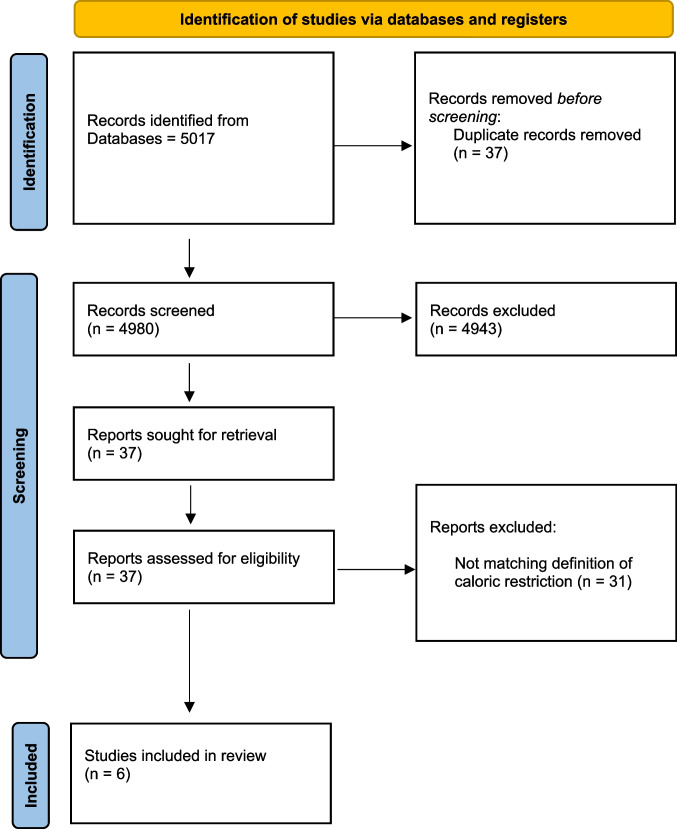
PRISMA flow-chart

The study selection process is described in Fig. 1. The electronic search initially identified 4980 articles. After the screening based on titles and abstract, 37 articles were selected for the full‐text analysis (Cohen’s kappa inter‐examiner agreement: 0.90). Of these, 6 articles were eligible to be included in the systematic review (Cohen’s kappa inter‐examiner agreement: 0.95). The main reason for exclusion was the fact that the majority of the studies did not follow the definition of caloric restriction. Hence, four of the included studies were animal studies [23–26], and the other two were human studies [15, 16].

### Description of animal model studies

The main characteristics of included animal model studies are summarized in Table 1. Three out of four studies were performed by the same research team [23–25] in rhesus monkeys that were housed at the National Institutes of Health Animal Center (Poolesville, MD, USA) for a previous research, and underwent a long-term diet (13–17 years) to assess the effect of caloric restriction on ageing [27]. The monkeys lived in a controlled environment and were continually monitored with regard to health status. A standard high-fibre diet, formulated by the National Institutes of Health, was provided [27]. Daily calorie intake was reduced by 30% in the caloric restriction group, and all animals (including the normal diet group) had their diet enriched with minerals and vitamins in addition to fresh fruit once a week. In one study [23], after 1, 2, and 3 months of ligature induced periodontitis, authors found that monkeys under the diet showed less gingivitis, bleeding on probing (BoP), probing depth (PD), and clinical attachment level (CAL) compared to the ad libitum diet group. Another study [24] that adopted the same dietary intake in the same type of monkey showed a gender-specific differentiation of responses oriented towards a potentially destructive inflammatory response in males and a protective adaptive immune response in females.

**Table 1:** Description of animal studies

Author and year	Subject	Follow-up	Type of intervention/periodontal status	Treatment test group	Treatment control group	Outcomes	Synthesis of results
Branch-Mays et al. 2008 [23]	55 monkeys	Baseline, 1-,2- and 3 months	Ligature-induced periodontitis	Caloric restriction: 30% less + Minerals and vitamins up to 100% daily + Fresh fruit once a week	Diet ad libitum	PD, CAL, BoP, GI and PI	Significantly lower PD, CAL, BoP and GI in test compared with controls
Ebersole et al. 2008 [24]	83 monkeys	13–17 years	No periodontal intervention	Caloric restriction: 30% less + Minerals and vitamins up to 100% daily + Fresh fruit once a week	Diet ad libitum + Fresh fruit once a week	CRP, fibrinogen, haptoglobin, alpha-1-AT and alpha-1-AG	Increased systemic acute phase reactants: alpha-1-AG and haptoglobin in males but were not affected by CR diet Increased serum IgG antibody responses to *Fusobacterium nucleatum* by CR diet in females only
Reynolds et al. 2009 [25]	81 monkeys	13–17 years	No periodontal intervention	Caloric restriction: 30% less + Minerals and vitamins up to 100% daily + Fresh fruit once a week	Diet ad libitum + Fresh fruit once a week	PD, CAL, IL-8, IgG and beta-glucuronidase	Increased PD and CAL, in males of the control group compared with females Decreased PD in males of the test group compared with the ones of the control group. Decreased IL-8, IL-1b, and beta-glucuronidase in GCF in test males compared with control males Decreased IgG antibody response
Wulansari et al. 2018 [26]	36 mice	4 weeks	Ligature-induced periodontitis	IF: 24 h without eating followed by 24 h of free access to food (15 cycles) PF: 48 h without eating followed by 5 days of free access to food (4 cycles) Food given or denied at 5 pm Water given ad libitum	Diet ad libitum	Bone loss and bone regeneration	Decreased bone loss and increased bone regeneration in both fasting groups compared with the non-fasting group

Abbreviations: *PD*, probing depth; *CAL*, clinical attachment level; *BoP*, bleeding on probing; *GI*, gingival index; *PI*, plaque index; *CRP*, C-reactive protein; *IgG*, immunoglobulin G; *CR*, caloric restriction; *IL-1/8*, interleukin-1/8; *IF*, intermittent fasting; *PF*, periodic fasting; *GCF*, gingival crevicular fluid

A further study [25] with the same methodology reported that test male monkeys had a significantly lower IgG antibody response and lower levels of Interleukin-8 (IL-8) and β-glucuronidase in gingival crevicular fluid (GCF) compared to control males, whereas the immune-inflammatory response of females was not affected.

A more recent mice model study [26] used two different fasting approaches in experimentally induced periodontitis compared to ad libitum diet (no specific information regarding the food intake were provided by authors). Intermittent fasting (every other day) and prolonged fasting (2 days fasting and 5 days normal food intake) were followed for 4 weeks. Both fasting regimen groups experienced less of bone loss (recorded using Micro CT analysis) compared to normal diet group at both the ligature-periodontitis sites and the contralateral side where the physiological bone loss usually happens. In addition, bone marrow cells from the femurs of the fasting groups produced more mineralized nodules than the bone marrow cells of the non-fasting groups when extracted and tested in vitro.

### Description of human studies

The main findings of human studies included are summarized in Table 2.

**Table 2:** Description of human studies

Author and year	Study	Subject	Follow-up	Type of intervention/periodontal status	Systemic condition	Treatment test group	Outcomes	Synthesis of results
Park et al. 2015 [15]	Cohort study	53 patients 41 obese patients 12 normal-weight patients	1 month	Healthy (no PPD > 3.5 mm)	Obese/normal weight	Low salt-low fat diet: ≤ 1300 kcal/day + 2 h of aerobic exercise + 3 h of weight training	BoP, GI and PI (at 1st and 27th days), CRP, GCF periodontal biomarkers, LDL, HDL, triglycerides in serum	Decreased levels of GCF (IL-b, MMP-8, MMP-9) in obese patients compared with the normal weight group Increased levels of IL-b in GCF, LDL, and body mass index in the obese group
Pappe et al. 2021 [16]	Prospective cohort study	36 patients	4 months	Periodontal disease (moderate to severe for women, PSI of 3 or 4; severe for men, PSI of 4)	Metabolic syndrome (MetS)	300–500 kcal/day + Diet/lifestyle programme	PSI, BoP, GCF, PI, CRP, BP, WC, FGLU, TRG, HDL and HbA1c	Significantly decreased levels of GCF and decreased BoP only in women No differences in men

Abbreviations: *PPD*, probing pocket depth; *CAL*, clinical attachment level; *BoP*, bleeding on probing; *GI*, gingival index; *PI*, plaque index; *CRP*, C-reactive protein; *IgG*, immunoglobulin *G*; *IL-b*, interleukin-b; *GCF*, gingival crevicular fluid; *LDL*, low-density lipoprotein (cholesterol); *HDL*, high-density lipoprotein (cholesterol); *PSI*, periodontal screening index; *BP*, blood pressure; *WC*, waist circumference; *FGLU*, fasting glucose; *TRG*, triglycerides; *HbA1c*, haemoglobin A1c: *MMP-8/9*, metalloproteinases-8/9

A cohort study conducted in a periodontally healthy Korean population [15] adopted a 4-week weight control intervention in young obese and normo-weight people. This intervention consisted of a low salt-low fat diet (≤ 1300 kcal/day) combined with 2 h of aerobic exercise and 3 h of weight training every day. No oral hygiene instructions were provided at any time. The authors observed a reduction of matrix metalloproteinase-8 (MMP-8), matrix metalloproteinase-9 (MMP-9) and interleukin-1β (IL-1β) in the GCF of the obese people, whereas no significant changes were observed in the normo-weight subjects. However, gingival index (GI) and BoP did not significantly decrease for both obese and normo-weight patients.

Another cohort study [16], carried out in patients with metabolic syndrome (MetS), used an intensified 2-week multimodal fasting, diet and lifestyle programme, consisting of a daily calorie intake limited to 300–500 kcal for a minimum of 4 days up to 10 days depending on the patient’s systemic health and well-being. Investigators found that in women with moderate to severe periodontitis (periodontal screening index (PSI) score of 3 or 4), a reduction of BoP and GCF volume was observed immediately after fasting. In contrast, no significant improvements were noticed in men with severe periodontitis (all with PSI score of 4). In addition, the authors mentioned that they intentionally did not provide any oral hygiene instructions or periodontal treatment during the whole study period since they wanted to assess whether there was a sole effect of fasting on periodontal inflammation. Thus, periodontal treatment was performed after the last follow-up, at 4 months [16].

### Sensitivity analysis based on risk of bias

According to the SYRCLE scale for animal studies, all studies presented an unclear risk of bias (Supplementary material). Newcastle Ottawa System revealed that the two cohort studies [15, 16] had a low score of 3 out of 9 stars that correspond to a high risk of bias (Supplementary material).

## Discussion

This systematic review assessed the evidence for the potential effect of different dietary restrictions on the periodontium. The analysed studies collectively showed that certain dietary restrictions might have the potential to reduce the local and systemic hyper-inflammatory state in periodontal patients. Besides, promising results demonstrated that dietary restriction may also impede periodontal disease progression. However, the evidence so far is very limited.

The last decades have brought a clearer understanding about the effect of diet on a whole series of human diseases and conditions. Several studies on the association of dietary intervention and periodontitis have also been published [9–13].

Very interestingly, caloric restriction and prolonged fasting are considered as a robust physiologic stimulus which causes a mild-to-moderate biological stress that, in turn, leads to numerous endocrine and neurobiological responses from systemic levels up to molecular signalling pathways [28]. As summarized by several narrative articles [28–30], dietary restriction might (i) increase DNA repair leading the cells to a state of repair and maintenance (stress-resistance hypothesis); (ii) decrease reactive oxygen species preventing damage to DNA, lipids and proteins (oxidative stress hypothesis); (iii) decrease insulin and glucose circulating levels leading to less cell division and more maintenance/repair (circulating glucose-insulin hypothesis); and (iv) stimulate cell growth and proliferation by decreasing the growth hormone/insulin like growth factor-1 signalling (IGF-1 hypothesis).

Even though fasting has been used by several populations since ancient times, the modern approaches involving fasting regimens have been developed following the principles of a periodic fasting, that is a diet that aims at simulating a real fasting and that hence is called “fasting mimicking diet” (FMD), which is applied for 4–7 days once every 15–30 days or less. The FMD consists of 30–50% of the normal caloric intake and a composition which mimics the effects of water only fasting on a number of markers, consumed for 4–7 consecutive days, followed by one transitioning day before returning to the ad libitum feeding [14, 31]. This periodic fasting was tested on humans and showed very promising effects in terms of reduction of risk factors for ageing, diabetes, autoimmunity, cardiovascular disease, neurodegeneration and cancer [14].

A pre-clinical study included in this review reported that, after inducing the periodontitis, caloric restriction might dampen the periodontitis inflammatory response in monkeys [23]. Several (pre-clinical) studies showed that low-calorie diet decreased the inflammation by reducing the release and effects of pro-inflammatory mediators [32, 33]. As reported by Ebersole et al., periodontal healthy male monkeys (in this project periodontitis was not experimentally induced) seemed to be more affected by periodontitis and less responsive to the diet [24]. Other studies demonstrated some inherent gender-based variations in levels of immunoglobulins [34] and host response biomarkers [35]. In addition, Reynolds and coworkers confirmed that, without inducing periodontitis, male monkeys presented an increased risk of developing periodontitis compared with females, and that some gender-related differences exist in modulatory effects of caloric restriction and local innate immune response [25]. Furthermore, an investigation in mice [26] found that two fasting approaches (intermittent and prolonged) led to less bone loss compared to mice that were fed ad libitum and, surprisingly, favoured bone formation (higher regeneration activity upon cessation of the experimental periodontitis model) at both sites where periodontitis was ligature-induced and at the control side. Other studies previously showed that fasting might prevent physiological bone loss in a periodontitis mouse model [36], and during ageing [37], in particular, prolonged fasting promotes regeneration in the hematopoietic and immune system [38], in the pancreas [39], in the spinal cord [40], and in the gut [41] as well as attenuates cerebral ischemic injuries [42], whereas intermittent fasting has the potential to improve cardiac function and survival from myocardial infarction [43].

Intriguingly, the existing human evidence partially confirm these pre-clinical findings. In fact, Pappe et al. adopted a fasting approach in MetS subjects with advanced periodontal disease and highlighted that, without providing any periodontal treatment, a short-term reduction in BoP and GCF volume occurred [16], albeit only in women. Furthermore, patient-reported outcome measurements (PROMs) revealed that all patients had no major adverse events after the fasting regime. Numerous studies demonstrated that dietary intervention might alter the immune response and/or homeostasis [44, 45]. Park et al. used a caloric restriction approach in obese and normo-weight periodontally health patients and revealed that the amount of the periodontal biomarkers in GCF was significantly reduced in absence of any periodontal treatment [15]. Nevertheless, a limitation of this study is that no PROMs were evaluated. A recent study adopting a periodic fasting approach (FMD) reported that such regimen might provoke some mild to moderate symptoms including fatigue, weakness and headaches but no severe side effects [31].

While at present the evidence is lacking, should an effect of dietary restriction on periodontal outcomes be proven, dietary regimes could be associated to mechanical periodontal therapy in order to improve clinical outcomes and potentially reduce systemic inflammation. In the clinical reality, complete fasting may be difficult to achieve. Therefore, an interesting solution to improve compliance might be to adopt a fasting mimicking diet approach (e.g., few days of fasting mimicking diet in few cycles per year) [31]. Notably, the recent guidelines proceeding from the European Federation of Periodontology do not strongly support the continuous caloric restriction regimen and, more in general, lifestyle changes due to the lack of a robust evidence [46].

Several limitations are present in the present review. First, the limited amount of the studies that was included. Second, the majority of them were animal studies, and three out of four were conducted by the same research group [23–25]. Further, the two human studies were cohort or case–control studies with only 4 months of follow-up period, and, lastly, high/unclear risk of bias was detected in the majority of the included studies. In detail, concerning animal studies, there was not enough information to answer to the questions of the SYRCLE tool (e.g., allocation concealment, blinding, random housing and random outcome assessment), whereas regarding the human studies, the main sources of bias are derived from the lack of information related to the independent or blind assessment, lack of comparability and inadequate (short) follow-up period. Future pre-clinical studies should be more precise in terms of blindness and randomization. Investigations on human populations should ideally be randomized controlled trials with a consistently longer follow-up period (at least 6 to 12 months).

In conclusion, to the best of our knowledge, this is the first systematic review that assesses the possible effect of dietary restrictions on periodontal disease. Overall, despite the paucity of the existing studies and some controversial but promising results, the present review concludes that some dietary/caloric restriction approaches may have the potential to affect periodontal conditions by reducing the local and systemic inflammation and by improving clinical parameters. In the search for adjunctive tools to improve periodontal outcomes and based on the promising evidence so far, there is an urgent need of methodologically robust studies that could evaluate dietary restrictions as an adjunctive therapy in patients with periodontal disease, assessing its potential effects on clinical and local/systemic outcomes and patient-reported outcome measurements, in the short- and long-term period.

## Supplementary Information

Below is the link to the electronic supplementary material.

Supplementary file1 (PDF 45 KB)

## Data Availability

The datasets used and/or analyses during the current study are available from the corresponding author on reasonable request.
