# Supplementary material for: Could dietary restrictions affect periodontal disease? A systematic review
Source: Clin Oral Investig. 2023 May 18;27(8):4107–16. doi: 10.1007/s00784-023-05052-9 (PMC10415456; doi:10.1007/s00784-023-05052-9)
Supplement: Supplementary file 1 — Supplementary file1 (PDF 45 KB) [file 784_2023_5052_MOESM1_ESM.pdf]

Supplementary Tables

Table S1. Risk of bias assessment following the SYRCLE scale for animal studies

	Selection bias			Performance bias		Detection bias		Attrition bias	Reporting bias	Other
**Study**	**Item 1**	**Item 2**	**Item 3**	**Item 4**	**Item 5**	**Item 6**	**Item 7**	**Item 8**	**Item 9**	**Item 10**
**Branch-Mays et al. 2008 [21]**	Unclear	Unclear	Unclear	Unclear	Unclear	Unclear	Unclear	Unclear	Yes	Unclear
**Ebersole et al. 2008 [22]**	Unclear	Unclear	Unclear	Unclear	Unclear	Unclear	Unclear	Unclear	Unclear	Unclear
**Reynolds et al. 2009 [23]**	Unclear	Unclear	Unclear	Unclear	Unclear	Unclear	Unclear	Unclear	Yes	Unclear
**Wulansari et al. 2018 [24]**	No	Unclear	Unclear	Unclear	Unclear	Unclear	Unclear	Unclear	No	Unclear

Table S2. Risk of bias assessment following the Newcastle Ottawa Scale

	Selection				Comparability	Outcome			
**Study**	**Item 1**	**Item 2**	**Item 3**	**Item 4**	**Item 5**	**Item 6**	**Item 7**	**Item 8**	**Total score**
**Partk et al. 2015 [13]**	0	1	1	0	0	0	0	1	**3**
**Pappe et al. 2021 [14]**	1	1	1	0	0	0	0	0	**3**
